# Increased Circulating Levels of Vitamin D Binding Protein in MS Patients

**DOI:** 10.3390/toxins7010129

**Published:** 2015-01-13

**Authors:** Arturo Ottavio Rinaldi, Isabella Sanseverino, Cristina Purificato, Antonio Cortese, Rosella Mechelli, Silvia Francisci, Marco Salvetti, Enrico Millefiorini, Sandra Gessani, Maria Cristina Gauzzi

**Affiliations:** 1 Department of Hematology, Oncology and Molecular Medicine, Istituto Superiore di Sanità, Rome 00161, Italy; E-Mails: artu.rinaldi@gmail.com (A.O.R.); asis83@yahoo.it (I.S.); cristina.purificato@iss.it (C.P.); sandra.gessani@iss.it (S.G.); 2 Multiple Sclerosis Center, Sapienza University of Rome, Rome 00161, Italy; E-Mails: corteseanto@libero.it (A.C.); enrico.millefiorini@uniroma1.it (E.M.); 3 Centre for Experimental Neurological Therapies (CENTERS) Department of Neurosciences, Mental Health and Sensory Organs Sapienza University, S. Andrea Hospital-site, Rome 00161, Italy; E-Mails: rosella.mechelli@uniroma1.it (R.M.); marco.salvetti@uniroma1.it (M.S.); 4 National Center for Epidemiology, Surveillance and Health Promotion, Istituto Superiore di Sanità, Rome 00161, Italy; E-Mail: silvia.francisci@iss.it

**Keywords:** vitamin D binding protein, albumin, relapsing/remitting multiple sclerosis, beta-interferon, immunology, vitamin D

## Abstract

Vitamin D (vitD) low status is currently considered a main environmental factor in multiple sclerosis (MS) etiology and pathogenesis. VitD and its metabolites are highly hydrophobic and circulate mostly bound to the vitamin D binding protein (DBP) and with lower affinity to albumin, while less than 1% are in a free form. The aim of this study was to investigate whether the circulating levels of either of the two vitD plasma carriers and/or their relationship are altered in MS. We measured DBP and albumin plasma levels in 28 MS patients and 24 healthy controls. MS patients were found to have higher DBP levels than healthy subjects. Concomitant interferon beta therapy did not influence DBP concentration, and the difference with the control group was significant in both females and males. No significant correlation between DBP and albumin levels was observed either in healthy controls or in patients. These observations suggest the involvement of DBP in the patho-physiology of MS.

## 1. Introduction

Multiple sclerosis (MS) is a neuroinflammatory and autoimmune disorder characterized by a progressive demyelination of axons of the central nervous system (CNS) and neuronal cell degeneration. A complex interplay between genetic and environmental factors contributes to the disease, and vitamin D (vitD) deficiency is currently considered a main environmental factor in MS etiology [1,2]. Low plasma levels of 25-hydroxyvitaminD (25(OH)D), the main circulating vitD metabolite, have been associated with a higher MS risk and a more severe clinical course [3,4]. Higher levels of circulating 25(OH)D were correlated with reduced relapse risk [5,6,7], and a recent large longitudinal prospective study showed that higher serum 25(OH)D levels robustly predicted a lower degree of MS activity and progression [8].

VitD and its metabolites are hydrophobic and circulate mainly bound to serum proteins. More than 85% of circulating 25(OH)D and 1,25(OH)_2_D (the bioactive vitD metabolite) is tightly bound to vitamin D binding protein (DBP). However, DBP is in large molar excess over its hormonal ligands, such that only 5% of blood DBP is bound to a vitD metabolite. Indeed, DBP can also bind with high affinity globular actin (G-actin). Thus, by sequestering G-actin released into the circulation upon tissue/cell damage or necrosis, DBP participates to the organism’s actin scavenging system [9]. This scavenging role of DBP may be vital, since free G-actin in the plasma tends to polymerize into long filaments, a condition that triggers disseminated intravascular coagulation if not rapidly resolved. Finally, DBP can enhance the chemotactic activity of complement 5a (C5a) and contributes to inflammation by being the precursor of macrophage activation factor (MAF), which derives from DBP through the stepwise modification of its sugar moiety [10].

Whether the DBP-bound fraction of vitD metabolites is biologically active is still a matter of debate. According to the most acknowledged “free hormone hypothesis” only free metabolites can cross the cell membrane and constitute the bioavailable hormone pool, while DBP mainly functions as a reservoir for their systemic delivery [11]. This hypothesis was challenged by the discovery of endocytic receptors that transport DBP-vitD complexes inside target organs [12] and are essential for the renal metabolism of vitD. Of note, DBP-dependent transport mechanisms are also thought to contribute to vitD access to the CNS [13]. Conversely, within the immune system, DBP was reported to inhibit the action of 25(OH)D3 and 1,25(OH)_2_D3 on APC [14,15] and to inhibit the conversion of 25(OH)D3 into 1,25(OH)_2_D3 by T cells [16].

Although the vast majority of vitD metabolites bind preferentially DBP, a small fraction (12%–15%) can also bind albumin, although with lower affinity, and less than 1% are in a free form [17,18]. One current hypothesis is that the free and albumin-bound pools are responsible for delivering 25(OH)D to the cell, with the DBP-bound 25(OH)D acting as a systemic reservoir [19].

Here, we measured circulating levels of the two vitD plasma carriers, DBP and albumin, and explored their potential correlation, in MS patients and healthy controls. Whether gender or IFNβ therapy influenced these parameters was also investigated. We found significantly higher plasma DBP levels in patients during phases of clinical remission, when compared with healthy subjects.

## 2. Results

**Figure 1:**
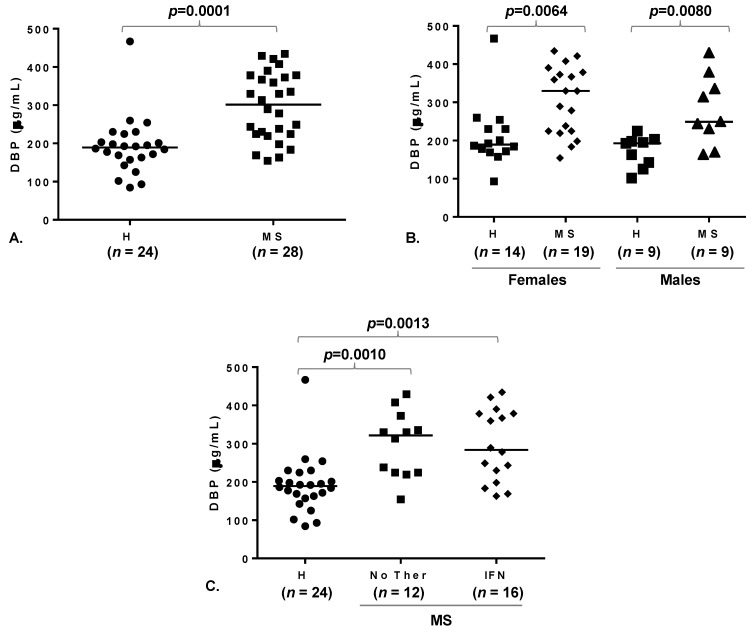
Plasma vitamin D binding protein (DBP) is increased in multiple sclerosis (MS) patients. Dot plots of plasma DBP concentration in MS patients and healthy controls. Horizontal lines indicate median values. (**A**) Comparison of healthy controls with MS patients. (**B**) Subject stratification by gender. Information about gender was missing for one healthy donor, which was excluded from this analysis. (**C**) Patient stratification by therapy. H, healthy donors; MS, MS patients (all in the remission phase of the disease); IFN, patients undergoing IFNβ therapy; No Ther, patients not undergoing any therapy. *p* value ≤0.05 were considered to reflect statistical significance.

We first compared plasma DBP levels in healthy controls (H) and MS patients. All patients were in the stable phase of the disease. Plasma DBP concentration was significantly higher in patients than controls (Figure 1A). Upon data stratification by sex (Figure 1B), both female and male patients presented higher DBP levels than healthy controls, while no significant gender difference was observed in either group. Comparable DBP levels were found in the subgroup undergoing IFNβ therapy (IFN) and the therapy-free one (No Ther), both displaying increased levels compared to controls (Figure 1C).

**Figure 2:**
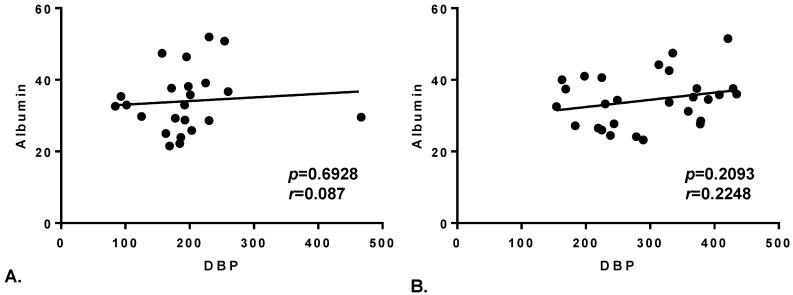
Correlation between plasma DBP and albumin. Linear correlation between plasma DBP and albumin in healthy controls (panel **A**) and MS patients (panel **B**). The *p* value and the Pearson’s correlation coefficient (*r*) are indicated.

The same analysis was performed on plasma albumin concentration. No significant differences among any group were present (Figure S1). Pearson’s correlation coefficients were calculated to assess the relationship between the two plasma carriers of vitD metabolites and whether this was influenced by the presence of the disease (Figure 2). No such correlation was found in healthy controls or in MS patients (Figure 2A,B) with *r* equal to 0.087 and 0.2248 respectively.

## 3. Discussion

Compelling evidence supports the association between low systemic levels of vitD and MS risk and disease activity [1,2], while only scattered data are available on DBP plasma concentrations in MS patients [20,21,22]. To the best of our knowledge, no previous study explored the possible correlation between DBP and albumin plasma levels. Yet, vitD protein carriers occupy a key node in the regulation of vitD access to target tissues [18].

We report here that, during the stable phase of the disease [23], MS patients present significantly higher plasma DBP concentration when compared with healthy subjects. An up-regulation of blood DBP was previously observed also in pediatric MS [20], while two studies reported no significant alterations in blood DBP levels in MS patients [21,22]. The apparent discrepancy may relate to differences in the control group that, in one study, included patients with other neurological diseases [21] and/or to differences in antibody specificity among commercially available immunoassays for DBP. Of note, two different DBP immunoassays has been recently compared in one study [24] and clearly shown to give different results, thus pointing to the urgent need of DBP measurement standardization [19,24]. Given the multifaceted activities of DBP, an alteration of its circulating levels may impact MS pathophysiology in different ways. One is by modifying vitamin D bioavailability. With the present knowledge we cannot anticipate whether vitD bioavailability is enhanced or impaired in remitting MS patients as a consequence of their higher blood DBP levels. However, we believe that our data add a further element of complexity to the assessment of the vitD status of MS patients. Indeed, the question of the standardization of meaningful measures of vitamin D status (*i.e.*, whether it can be solely 25(OH)D concentration, or should incorporate other indices such as DBP or albumin) is an emerging health need [19,25]. The accurate definition of patient vitD status may be particularly critical when evaluating the efficacy of vitD supplementation in RRMS in clinical trials [26,27]. Interestingly, data obtained in an experimental allergic encephalomyelitis model of MS in rats suggest that the benefits of vitD supplementation, which delayed onset and diminished severity of the disease, are lost when DBP is up-regulated [28].

Any condition which may alter DBP levels, including concomitant disease modifying therapies, could in principle influence the vitD therapeutic effect itself. In this regard, our data showing no major effect of IFNβ therapy on DBP blood levels are of relevance, as vitD is on trial also as add-on treatment to RRMS patients undergoing IFNβ therapy [26].

MS incidence rates are 2–3 fold greater in women than in men, and gender differences in the biological effects of vitD metabolites were described [29]. We thus asked whether gender effects contribute to the altered DBP plasma levels of MS patients reported here. However, upon data stratification by sex, the difference with healthy donors remained significant in both females and males, while no gender difference was found in patients or in the control group. This suggests that the known enhancing effect of estrogens on DBP hepatic synthesis [30,31,32] may not be a major confounding factor in this study.

DBP properties other than that of vitD plasma carrier could be involved in MS pathogenesis. For example, it cannot be excluded that, through its activity as MAF precursor, DBP could foster neuroinflammation. Additionally, through its role as cofactor enhancing C5a-mediated macrophage and neutrophil chemotaxis, higher DBP levels could favor immune cell infiltration into the CNS.

## 4. Materials and Methods

### 4.1. Subjects

Peripheral blood was collected in EDTA from 28 relapsing remitting MS patients [23] whose demographic and clinical characteristics are summarized in Table 1. At blood withdrawal, all patients were free of corticosteroid therapy and in the remission phase of the disease, as based on clinical features. Blood samples from 24 healthy volunteers, with a comparable gender and age distribution, were also collected. All blood donors gave their written informed consent to this observational study, approved by the Research Ethics Committee of the Istituto Superiore di Sanità.

**Table 1:** Demographic and clinical characteristics of MS patients.

No of subjects	28
Age (years; mean ± SD)	36 ± 8
Females/males (*n*)	19:9
EDSS ^1^ (mean ± SD)	0.98 ± 1.93
Disease duration (years; mean ± SD)	5.3 ± 3.8
No of subjects on IFN therapy ^2^	16

^1^ EDSS, Expanded Disability Status Scale; ^2^ Patients were undergoing IFNβ treatment for at least 1 month before inclusion in the study.

### 4.2. Biochemical Analysis

Plasma was obtained as the upper layer of whole blood centrifugation on ficoll gradient. Plasma concentrations of actin-free DBP and albumin were measured by commercial ELISA kits (DRG Diagnostics, Marburg, Germany and Cloud-Clone Corp., Houston, TX, USA, respectively) according to manufacturer’s instructions. All biochemical measures were performed in a single batch and a comparable number of patient and control samples was always assayed simultaneously in the same ELISA plate.

### 4.3. Statistical Analysis

Differences between groups were tested with the non-parametric Mann-Whitney two-sample statistic test for independent samples. Two-tailed *p*-values <0.05 were considered statistically significant. Correlations were tested with the Pearson correlation coefficient. Analyses were conducted using Stata/IC (Version 11.0 for Windows (32-bit), StataCorp LP 4905 Lakeway drive, College Station, TX, USA, copyright 1984–2009) and Graphpad (Version 6.05, GraphPad Software, Inc., La Jolla, CA, USA, 2014).

## 5. Conclusions

Our data, together with results from proteomic studies showing that DBP levels in the cerebrospinal fluid correlate with the MS course [33], point to a still under-recognized involvement of this protein in MS. They also highlight the need of better understanding whether and how DBP influences biological and clinical end points linked to the vitD status.

## Supplementary Materials

Supplementary materials can be accessed at: http://www.mdpi.com/2072-6651/7/1/0129/s1.
